# Hepatocellular Carcinoma in Chronic Viral Hepatitis: Where Do We Stand?

**DOI:** 10.3390/ijms23010500

**Published:** 2022-01-02

**Authors:** Francesco Paolo Russo, Alberto Zanetto, Elisa Pinto, Sara Battistella, Barbara Penzo, Patrizia Burra, Fabio Farinati

**Affiliations:** Gastroenterology/Multivisceral Transplant Unit, Department of Surgery, Oncology, and Gastroenterology, Padova University Hospital, 35128 Padova, Italy; francescopaolo.russo@unipd.it (F.P.R.); alberto.zanetto@yahoo.it (A.Z.); pintoelisa93@gmail.com (E.P.); sarabattistella93@gmail.com (S.B.); barbara.1992.bp@gmail.com (B.P.); burra@unipd.it (P.B.)

**Keywords:** hepatocellular carcinoma, HBV, HCV, survival, cirrhosis

## Abstract

Hepatocellular carcinoma (HCC) is one of the major causes of cancer-related death. Although the burden of alcohol- and NASH-related HCC is growing, chronic viral hepatitis (HBV and HCV) remains a major cause of HCC development worldwide. The pathophysiology of viral-related HCC includes liver inflammation, oxidative stress, and deregulation of cell signaling pathways. HBV is particularly oncogenic because, contrary to HCV, integrates in the cell DNA and persists despite virological suppression by nucleotide analogues. Surveillance by six-month ultrasound is recommended in patients with cirrhosis and in “high-risk” patients with chronic HBV infection. Antiviral therapy reduces the risks of development and recurrence of HCC; however, patients with advanced chronic liver disease remain at risk of HCC despite virological suppression/cure and should therefore continue surveillance. Multiple scores have been developed in patients with chronic hepatitis B to predict the risk of HCC development and may be used to stratify individual patient’s risk. In patients with HCV-related liver disease who achieve sustained virological response by direct acting antivirals, there is a strong need for markers/scores to predict long-term risk of HCC. In this review, we discuss the most recent advances regarding viral-related HCC.

## 1. Introduction

Hepatocellular carcinoma (HCC) is the most frequent type of primary liver cancer and the second most frequent cause of cancer-related death worldwide [1]. In approximately 90% of patients, HCC is associated with cirrhosis [1]. Although the burden of alcohol- and NASH-related HCC is growing [2], chronic viral hepatitis remains a major cause of liver cancers worldwide [3,4].

In this review, we discuss the current knowledge regarding mechanisms of viral-related carcinogenesis, risk factors, and epidemiology of viral-related HCC, and strategies for surveillance in patients at risk (first section). We will also discuss how antiviral therapy reduces the risk of HCC in patients with chronic viral hepatitis and include specific consideration regarding the emerging field of immunotherapy in these patients (second section).

## 2. Viral Hepatitis and HCC: Epidemiology and Risk Factors

Epidemiology of HCC is significantly changing [5]. In the traditional “high-risk” areas (i.e., south-east Asia), incidence and mortality are progressively decreasing. By contrast, both incidence and mortality are increasing in North America and in some (but not all) European countries [6]. These trends likely reflect different exposure to “traditional” (i.e., HBV) and “new” (i.e., non-alcoholic fatty liver disease) risk factors and difference in surveillance [7]. In our region (Italy), recent studies based on the ITA.LI.CA database clearly demonstrate that the number of viral-related HCC not only is significantly and progressively decreasing since the late 1990s, but is also expected to further decrease in the next future [8,9].

Numerous non-viral related factors such as alcohol-related liver disease, non-alcoholic steatohepatitis (NASH), diabetes, and non-alcoholic fatty liver disease (NAFLD) are becoming progressively more relevant for the development of end-stage liver disease and HCC [1,10]. However, chronic hepatitis C (HCV) and chronic hepatitis B (HBV) viruses remain of primary importance [6].

The European Association for the Study of Liver (EASL) guidelines recommend vaccination against hepatitis B in all new-born and high-risk populations to reduce the spread of HBV infection [4]. In fact, chronic hepatitis B infection (CHB) is the major risk factor associated to development of HCC worldwide and, importantly, HCC may arise in patients with HBV chronic infection *without cirrhosis* [11]. In one recent study including 8539 patients from the Veterans Administration, 317 developed HCC, of whom 30 (9.5%) did not have any evidence of cirrhosis at time of HCC diagnosis [12]. Compared to HCC patients with cirrhosis, these patients were more likely to be African American or Asian, have a family history of HCC, and hypertension, which suggests that these factors may be helpful to select patients with CHB without cirrhosis at higher risk in whom to consider for surveillance.

Additional and synergistic risk factors for development of HCC in CHB are patient-related (male sex, older age, Asian or African ethnicity, history of HCC within the family), viral-related (high viral replication, HBV genotype, longer duration of infection, co-infection with HDV, HCV or HIV), clinical-related (presence of cirrhosis), or environment-related (exposure to aflatoxin, history of alcohol abuse and smoking) [1,4]. Treatment with nucleotides analogues (NA) and suppression of HBV replication is the most important factor in determining the reduction in HCC risk (see below) [13,14,15]. In patients receiving NA, older age, cirrhosis, male sex, platelets number, liver stiffness, and diabetes are additional risk factors that are associated with increased risk for HCC and may be considered to stratify patient’s risk [16,17,18]. The Cirrhosis, Age, Male sex, Diabetes (CAMD) score was created to estimate the risk of HCC in Asian patients treated with continuous NA therapy (tenofovir or entecavir) [19].

Hepatitis C virus (HCV) infection is another leading cause of chronic liver disease. Prior to introduction of direct acting antiviral therapy (DAAs) therapy [20], there were approximately 71 million chronically infected individuals worldwide [21]. Achievement of sustained virologic response (i.e., virological cure, SVR) is associated with a significant reduction in the risk of HCC [3]. A previous meta-analysis including data from cohorts treated with interferon-based therapy demonstrated a reduction by more than 70% in the risk of HCC occurrence after SVR, regardless the severity of baseline liver fibrosis [22]. Remarkably, there was a significant reduction also in patients with cirrhosis though these patients remained at significant risk (<1.5% [0.3–2.4%]) [23,24]. The development of the new DAAs has changed the scenario of HCV-related HCC and numerous independent studies confirmed that patients who achieve SVR after DAAs have a significantly reduced risk of HCC [25,26] (see below).

Additional factors that may influence the risk of residual HCC in patients with HCV infection (both active and treated patients) are older age, male sex, Hispanic ethnicity, diabetes and obesity, smoking, HCV genotype 3, alcohol abuse, and coinfection with HIV or HBV [27,28,29]. There are increasing data indicating that a combination of such factors may improve risk stratification and help to identify patients who remain at risk of HCC despite SVR (see below).

Hepatitis E virus (HEV), particularly genotype 3 and 4, is being increasingly recognized as a potential cause for liver disease and cirrhosis in immunocompromised patients [30]. Preliminary data suggest that HEV-driven hepatic carcinogenesis is rare. However, with the increasing knowledge regarding complex clinical manifestations of HEV chronic infection and potential mechanisms of HEV-driven cell transformation, the question arises as to whether these patients are at increased risk of HCC [31]. Further studies on laboratory and clinical aspects of HEV-driven HCC are expected.

## 3. Viral-Related Hepatic Carcinogenesis

Hepatic carcinogenesis is a complex process in which genetic predisposition, cellular microenvironment, immune cells, and viruses play different but synergistic roles [32]. Three main mechanisms are involved in the development of HCC: persistent liver inflammation, oxidative stress, and deregulation of cell signaling pathways [32]. In general, oncogenic viruses do not lead to development of cancer per se, instead it is the interaction with host factors (i.e., dysregulation of the immune system) that leads to preneoplastic conditions and then cancer [33]. Chronic inflammation due to viral infection leads to a progressive alteration of immune cells, which causes an increased release ROS and proinflammatory cytokines within the liver niche and finally determines the remodeling of liver microenvironment [34]. Recently, it has also been suggested that alterations of hemostasis, particularly of platelets, could have a role in hepatocarcinogenesis [35,36]. Additional mechanisms that promote the induction of HCC in patients with HBV, HCV, and HBV-HDV infection are discussed below (Table 1) [37,38,39,40,41,42,43].

### 3.1. HBV

HBV is a partially double-stranded circular DNA virus and is able to integrate in hepatic cell’s DNA, thus leading to chromosomal rearrangements, genomic instability, and mutagenesis in proto-oncogenes and tumor suppressors [44]. This appears the main mechanism through which HBV may result in HCC in the absence of cirrhosis.

In patients with cirrhosis, development of HBV-associated HCC is multifactorial. Firstly, the X protein, expressed in the HBV genome, interacts with nuclear transcription factors and signal transduction pathways such as Raf, c-Jun, MAPK, NFκB, Jak-Stat, FAK, and protein kinase C pathways, as well as Src-dependent and phosphatiylinositol-3 kinase signaling cascades [45,46].

HBx causes hypermethylation or global hypomethylation of the DNA, leading to the silencing of tumor suppressor genes and chromosomal instability [47]. Moreover, it exerts both anti-apoptotic [48,49] and pro-apoptotic activity [50] and may increase the expression of TERT and telomerase activity [51]. Finally, the HBx protein is involved in the dysregulation of IGF-II11. All these processes result in uncontrolled cell growth and malignant transformation.

Among the most relevant deregulated pathways, the Wnt/FZD/β-catenin, PI3K/Akt/mTOR, insulin receptor substrate 1 (IRS1)/insulin-like growth factor 1 (IGF), and the Ras/Raf/mitogen-activated protein kinases (MAPK) pathways are the most important [40]. WNT is a stem cell regulator that, when binding with FZD and LRP, inhibits the destruction of β-catenin, which translocates to the nucleus and forms a transcriptionally active complex with the T-cell-specific transcription factor/lymphoid enhancer-binding factor (TCF/LEF). This results in the expression of WNT target genes, which, in turn, lead to uncontrolled liver cell proliferation and survival and, finally, to HCC development [52,53]. The upregulation of PI3K/AKT and Ras/ERK1/2 brings about the same effect of WNT activation through the overexpression of cyclin D1 and the activation of c-Myc and NFκB [54,55,56]. Finally, HBV exposes hepatocytes to FasL increasing apoptosis and the resulting compensatory regeneration, which may determine HCC [57,58,59]. HBV infection in Asian countries is frequently associated with aflatoxin exposure; in that case, HBV-related carcinogenesis is specifically characterized by development of mutations in the gatekeeper p53 tumor suppressor gene [60].

### 3.2. HDV

Processes such as immune response modification, epigenetic changes, or oxidative stress in the ER may also be related to HDV infection. In fact, HDV acts on the same pathways mentioned before (TGF-β, Smad3, STAT3, NFκβ), promoting cell survival, cell growth, and malignant transformation [61,62,63]. Furthermore, HDV evades IFN-α mediated immune response promoting cell survival [64].

### 3.3. HCV

Unlike HBV, HCV cannot integrate into human genome. Three major mechanisms are involved in HCV-related hepatocarcinogenesis: chronic inflammation, deregulation of immune response, and altered function of antigen-presenting cells [65].

The interaction between viral protein NS5A and cellular components [66] alters two main cellular functions such as protein synthesis (because activity of endoplasmic reticulum is shifted towards the synthesis of viral rather than cellular proteins) [67] and lipogenesis (which leads to accumulation of free fatty acid within the cytosolic space) [68]. In a vicious cycle, the function of endoplasmic reticulum is further damaged by the oxidative stress due to the accumulation of long chain fatty acids and cholesterol in the infected cells, which finally leads to activation of NFkB pathway [69,70]. Downstream to NFkB, there are the same pathways described for HBV-related carcinogenesis [65]: TERT, β-catenin, p53, Rb, chromatin remodeling/epigenetic modifications, hepatocyte differentiation, PI3K-mTOR pathway, and NRF2-kelck-like ECH-associated protein (KEAP), cancer stem cells, angiogenesis and RTKs. On top of that, HCV core and NS3 proteins can determine an increase of inflammatory cytokines, such as IFN-β, IL-1β, IL-6, TNF-α that may lead to malignant evolution [71,72].

## 4. Surveillance in Patients at Risk

Surveillance with six-month abdominal ultrasound is recommended in patients at risk for HCC [1]. This includes patients with cirrhosis, independent of etiology, and selected patients with chronic HBV infection (Asian males hepatitis B carriers over the age of 40s, Asian female hepatitis B carriers over the age of 50s and hepatitis B carriers with a family history of HCC) [1,4]. There is no advantage with a more intense screening strategy (i.e., every three months) [73], not even in patients at high risk for HCC [74]. By contrast, the prolongation of time interval from six months to one year is associated with a significantly increased risk of late diagnosis and death [75].

There is an ongoing debate regarding surveillance in patients with advanced liver fibrosis (F3). According to the EASL guidelines, ultrasound surveillance may be recommended in these patients based on the evaluation of individual risk factors [1]. By contrast, American guidelines do not recommend screening in F3, but only in patients with cirrhosis [76].

The incidence of HCC differs according to chronic liver disease etiology, clinical, pathological, and epidemiologic factors, and geographical distribution. Therefore, the identification of a single score system to predict the development of HCC is challenging.

The HCC risk scores for CHB are differentiated between treated and untreated patients. The majority of them are developed in Asian populations, but some are also validated in Caucasian cohorts (Table 2).

Among untreated patients, the first risk score to be proposed was the GAG-HCC score, whose formula is: 16 × sex (male = 1; female = 0) + age (in years) + 3 × HBV DNA levels (copies/mL in log) + 19 × core promoter mutations (mutant = 1; wild-type = 0) + 30 × cirrhosis (presence = 1; absence = 0). The score hazard ratio for the development of HCC is 1.07 (95% CI 1.05–1.08, *p* < 0.001), indicating that the risk of HCC development increases by 7% per one-point increase in GAG-HCC score [77]. The second was the CU-HCC score that included age (<50 or >50), albumin (≤35 or >35 g/L), bilirubin (> 18 or ≤18 umol/L), HBV DNA (≤4, 4–6 or >6 log), and cirrhosis (yes or no) to stratify patients into three groups (total score < 5, between 5 and 19, and >19) with progressively increasing risk of HCC (none in the first group, 26.8% in the second group, and 31.4% in the third group). Interestingly, the negative predictive value for “low risk” group by the CU-HCC score was 97.8%, indicating that HCC surveillance in this group may be interrupted [78]. A third score by Yang et al., called the REACH B score [79], includes age, male sex, alanine aminotransferase (ALT), HBeAg positivity, and HBV-DNA levels. The authors identified three distinct groups (≤5, 6–11, and ≥12) with significantly different risk of HCC (0.2, 3.3, and 47.4%, respectively). Finally, the RSW-HCC is another simple score elaborated in 538 patients with CHB and then validated in 3353 subjects from the REACH-B, GAG-HCC, and CU-HCC cohorts. This score includes age, gender, cirrhosis, and levels of AFP (between 4.1 and 20 microg/mL vs. >20 microg/mL). An RWS-HCC score > 4.5 indicates a significant risk of development of HCC over the next ten years with a sensitivity of 88.1% and a specificity of 83% [80].

PAGE-B score was the first score to be developed in Caucasian patients (treated) and includes age, gender, and platelet count. This score distinguishes three groups with a five years cumulative HCC incidence of 0, 3–4, and 16–17%, respectively. Importantly, this score has a 100% NPV in patients at low risk, thus potentially being useful to identify patients therefore not eligible for surveillance [81]. Recently, the REAL-B score was developed in a cohort of 8048 CHB. It includes male gender, alcohol use, cirrhosis, age, diabetes, AFP and platelet count to divide patients in three categories with an annual incidence rate of 0.09, 0.9, and 5.8% respectively [82].

Few risk scores are available in patients with chronic hepatitis C infection. In 2016, the ANRS CO12 CirVir, a study based on a multicenter cohort of 1323 CHC-related cirrhosis patients, proposed a risk score based on five variables to predict the development of HCC (age > 50 years, low platelet count, GGT > ULN, past excessive alcohol intake and absence of SVR) [83]. A Japanese study in 2020 proposed an even more simplified scoring system (0–2 points) based on level of AFP and age, with a 0.3, 6.2, and 18.3% incidence of HCC in patients with 0, 1, and 2 points, respectively. However, this score lacks an external validation [84]. Additional evidence is accumulating regarding how to identify patients who remain at higher risk of HCC after the achievement of SVR (see below). While it is unlikely that patients with cirrhosis can stop screening after virological cure, these scores may be helpful to identify patients with less advanced fibrosis (F2–F3) and with no additional risk factors (i.e., alcohol) in who the achievement of SBR abolishes the risk of future cancer.

**Table 2:** Published scores for the evaluation of HCC risk in patients with chronic HBV.

Name (Ref.)	Use of Antiviral Treatment	Patients (Number and Ethnicity)	Variables Included in the Score	Risk Categories (Scores)	Incidence of HCC According to Risk Category	NPV (%)
GAG-HCC [77]	No	820 Asian and Caucasian	Age, gender, HBV-DNA, cirrhosis	Low (<101) High risk (>101)	Not available	99% at 10 year
CU-HCC [78]	No	1055 Asian and Caucasian	Age, HBV-DNA, cirrhosis, bilirubin, albumin	Low (<5), Intermediate (5–20) High-risk (>20)	5 and 10-years HCC-free survival rates: Low: 98.3% and 97.1% Intermediate: 90.5% and 71.0% High: 78.9% and 67.7%	97% at 10 year
RSW-HCC [80]	No	538 Asian and Caucasian	Gender, cirrhosis, aFP	Low (<4.5) High-risk (>4.5)	Not available	98.8% at 10 year
PAGE-B [81]	Entecavir/ tenofovir	1325 Asian and Caucasian	Age, gender, platelet count	Low (<9), Intermediate (10–17) High-risk (>18)	Low: 0% at 5 years Intermediate: 3% at 5 years High: 17% at 5 year	100% 5 year
REAL-B [82]	Yes (not specified)	5365 Ethnicity not available	Age, gender, alcohol use, cirrhosis, alpha-fetoprotein, platelet count, diabetes	Low (<3) Intermediate (4–7), High-risk (8–13)	Low: < 1.38% at 5 years and <3.28% at 10 years Intermediate: <10.24% at 5 years and <22.82% at 10 years High: up to 90.37% at 5 years and up to 99.63% at 10 years	Not available

Legend: HCC: hepatocellular carcinoma, NPV: negative predictive value; AFP: alpha-fetoprotein.

In HBsAg-positive patients with cirrhosis, HDV infection increases the risk of HCC and liver-related mortality two- and three-fold, respectively [85,86]. A recent systemic review with meta-analysis found that the risk of HCC was two-fold higher in 6099 HBV/HDV co-infected patients than in 57,620 chronic HBV mono-infected patients [87]. Importantly, the magnitude of effect did not differ after adjustment for study design and quality, publication year and duration of follow-up, thus suggesting that the increase of HCC risk in HDV patients is real and significant [87]. Regarding risk factors, cirrhosis, HDV-RNA positivity, age > 50 years old, male gender, and BMI are independently predictive of HCC development in patients with CHB treated with NAs. Interestingly, in the subgroup of those with cirrhosis, HDV-RNA positivity remains independently predictive of HCC [88].

Whether specific strategies based on such risk factors may improve the detection of HCC in patients with HBV/HDV chronic infection is unclear. Among current guidelines for the management of HBV, some but not all recommend systematic screening for HCC in HDV patients. In fact, while the American Association for the Study of the Liver Guidelines indicate that HBsAg-positive patients with HDV coinfection should receive screening, independently of cirrhosis [89], European [4] and Asiatic [90] guidelines are not as stringent and favor an individualized approach. Incorporating new host factors such as positivity of HDV status in assessing the risk of HCC is an important area for future research [91]. In fact, pending authorization by the Medicine Agencies, new treatments for HDV will soon become available [92] and hopefully will help reduce the burden of HDV-associated HCC.

In conclusion, all patients with cirrhosis should undergo HCC surveillance regardless etiology of liver disease, sex, age, and origin. Whether and how ultrasound screening should be extended to patients without cirrhosis but with advanced liver fibrosis (F3) requires further investigation.

## 5. The Impact of Antiviral Therapy on the Risk of HCC in Patients with Chronic Viral Infection

In patients with chronic viral infection, antiviral therapy and suppression/cure of viral replication is associated with a significant reduction in the risk of HCC development and recurrence [1,3,4]. In this paragraph, we will discuss current strategies of antiviral therapy and control of oncological risks in patients with HBV- and HCV-related chronic liver disease.

### 5.1. Patients with HBV Chronic Infection

Treatment of HBV chronic infection includes pegylated interferon-alpha (Peg-IFN alpha) and oral nucleotide analogues (NAs). The NAs include lamivudine (LAM), adefovir dipivoxil (ADV), entecavir (ETV), telbivudine (TBV), tenofovir (TDF), and tenofovir alafenamide (TAF). Long-term administration of a NA with high-barrier to virological breakthrough (ETV, TDF, TAF) is the treatment of choice, regardless of the severity of liver disease [4]. This allows to achieve virological remission in virtually all patients with CHB and is associated with significant improvement of liver necroinflammation and fibrosis, which translates into reduced risk of HCC [93].

The reduction of HCC risk depends on the maintenance of persistent virological remission. In fact, the risk of HCC increases significantly in patients with previous history of virologic breakthrough (even when suppression of HBV replication is then re-achieved by a rescue therapy) [4]. In fact, lamivudine and adefovir, which are associated with a high risk of virological resistance, have been completely replaced by ETV and TFV as first-line treatment.

There is strong evidence that treatment with NAs reduces the risk of HCC in patients with CHB [94]. In a previous meta-analysis including >10,000 patients with CHB receiving NAs [95], the pooled rate of HCC per 100 person-years of follow-up was two-fold in patients with detectable HBV-DNA vs. patients with undetectable HBV-DNA higher (1.9 vs. 1 per 100 person-years), which indicates the long-term need for maintaining the suppression of HBV replication. Older patients, with cirrhosis, and persistently detectable HBV-DNA during therapy were at increased risk of HCC. Importantly, the same meta-analysis showed that the risk of HCC was ten-fold higher in cirrhosis vs. patients without advanced chronic liver disease (3 vs. 0.3% per 100 person-years). By contrast, the risk of HCC development was not different in compensated vs. decompensated patients (3.6 vs. 2.5 per 100 person years) [95]. Therefore, it is of primary importance to continue surveillance for HCC in patients with cirrhosis receiving NAs [1,4].

On the other hand, it is also important to underline that therapy with NAs significantly reduces the risk of HCC also in patients with cirrhosis. In a recent study, Su et al. [96] observed that the risk of HCC after four years of NAs was reduced by 60–70% (compared with untreated patients), and that the five-year cumulative incidence of HCC was 26.4% in untreated vs. 11.3% in treated patients. They also found that HBeAg positivity was associated with a significantly increased risk of HCC (both in patients with and without cirrhosis), and that antiviral therapy with ETV reduced the risk of HCC in both HBeAg-positive (HR: 0.39, 95% CI: 0.20–0.75) and in HBeAg-negative (HR: 0.40, 95% CI: 0.26–0.62) patients. Patients who had HCC during follow-up were older and had lower albumin and platelet count. On multivariate analysis, older age (HR: 1.06, 95% CI: 1.04–1.08), male gender (HR: 1.88, 95% CI: 1.20–2.96), HBeAg positivity (HR: 1.89, 95% CI: 1.23–2.92), baseline level of AFP level ≥ 7 ng/mL (HR: 1.93, 95% CI: 1.30–2.88), and lack of 1-year virological response (HR: 0.62, 95% CI: 0.40–0.98) were independent predictors of HCC [96].

Whether one NA is superior than another is unclear. No difference was found between ETV and TDF in one study including 1325 patients with CHB both with and without cirrhosis (annual incidence of 2.50 and 2.00% per year in cirrhosis treated with ETV vs. TDF, respectively) [97]. In patients without advanced fibrosis, the four cases of HCC were all observed in those treated with ETV. However, at multivariate analysis, the risk of HCC was comparable between ETV and TDF (HR 1.66, 95% CI 0.77–3.59, *p* = 0.199, and HR 1.49, 95% CI 0.67–3.32, *p* = 0.327 in cirrhosis and non-cirrhosis, respectively) [97]. Similar findings were reported by a larger and more recent cohort including almost 2000 patients with and without cirrhosis treated with ETV and TDF and followed for up to eight years (1-, 3-, 5-, 8-year cumulative incidence of HCC of 1.8%, 5.0%, 12.7%, 18.9% vs. 3.6%, 9.2%, 15.5%, 20.8 in cirrhosis, *p* = 0.4: 0.3%, 1.5%, 3.5%, 4.6% vs. 0.5%, 1.4%, 1.7%, 2.8%, *p* = 0.1 in non-cirrhosis) [98]. The risk of HCC during ETV and TDF treatment was also investigated separately in patients who were naïve vs. those with previous exposure to other NAs. While the overall risk of HCC risk was higher in experienced patients (as one would expect), the cumulative incidence of HCC was comparable between naive (1-, 3-, 5-, 8-year cumulative HCC rates: 0.7%, 1.9%, 5.4%, 6.6% vs. 1.4%, 4.0%, 5.7%, 6.8%; *p* = 0.7) and ETV and TDF experienced patients (1-, 3-, 5-, 8-year cumulative HCC rates: 0.6%, 3.2%, 5.4%, 11.5% vs. 1.6%, 3.7%, 6.3%, 9.9%; *p* = 0.894) [98].

There are other studies, all from Asia, suggesting that TDF may be associated with a lower risk of HCC than ETV [99,100]. In one cohort including 24,156 patients with chronic HBV infection, Choy et al. found that tenofovir was associated with a lower risk of HCC compared to entecavir [99]. Importantly, as this was confirmed by propensity score-matching and competing risk analyses (both overall and in patients with and without cirrhosis, separately analyzed), the authors concluded that this difference could be explained by the superior profile of tenofovir [99]. However, as the virological resistance profile was not independently predictive of HCC, the difference between the two groups was not entirely explained by the different antiviral potency.

Different models for stratification of HCC risk have been proposed in patients with CHB infection. All these scores combine various variables related to either HBV infection or demographics or severity of liver disease and, as discussed before, most of these scores have been created and validated in Asian countries [77,78,79].

The PAGE-B score, on the other hand, was the first score to be proposed for Caucasian patients treated with ETV or TDF [81]. The score includes age, gender, and platelets as independent risk factors for development of HCC and stratifies patients into three groups at different risks of HCC (low, medium, and high-risk, with a score of ≤ 9, 10–17, and ≥18, respectively). The five-year cumulative probability of HCC was 0, 3, and 17% in patients in the low, medium, and high-risk group, respectively. Two additional scores developed in Caucasians patients treated with ETV/TDF therapy for more than year were the CAGE-B and SAGE-B scores [101]. Interestingly, both scores have high negative predictive value and therefore may be used to identify patients with a very low risk of HCC in whom to consider interruption of surveillance.

In patients with chronic HBV infection in whom HCC has already arisen, antiviral treatment may have antitumoral effects and, in combination with treatment of HCC, improves patient survival [102]. In fact, it has been shown that replication of HBV has a role in determining HCC recurrence [103] and reducing post-operative survival [103,104].

In one study by Wong [15], NA therapy was associated with a 41% reduction in the risk of HCC recurrence after curative treatment of HCC and was associated with a 90% reduction in the risk of liver-related mortality and 78% reduction in overall mortality. Comparable findings were reported by other groups and confirmed that antiviral therapy with NA is independently protective of both early and late recurrence of HCC after treatments [105]. Importantly, antiviral therapy with NA was independently associated with improved survival also in patients receiving non-curative treatment, such as chemoembolization [106,107,108].

In conclusion, antiviral therapy significantly reduces the risk of HCC in patients with chronic HBV infection, including those with cirrhosis. Older age and presence of cirrhosis at baseline are independent risk factors for development of HCC. Multiple scores are available to determine the risk of HCC development in patients receiving antiviral therapy and may be considered to predict individual risk of cancer. In patients with HCC who undergo curative and non-curative treatment for HCC, the use of antiviral therapy is recommended to reduce the risk of HCC recurrence and improve patient’s survival.

### 5.2. Patients with HCV Chronic Infection

Eradication of HCV is associated with a significant reduction in the risk of HCC [3]. This was clearly demonstrated in patients treated by interferon (IFN)-based antiviral therapy [109,110,111]. In a previous analysis including data from 12 cohort studies, the achievement of sustained virological response (i.e., virological cure, SVR) by IFN therapy led to a four-fold reduction in risk of HCC, independently of the baseline severity of chronic liver disease [22]. In another long-term observational cohort, the ten-year cumulative incidence of HCC was significantly lower in patients with vs. without SVR (5.1 vs. 21.8%, respectively; *p* < 0.001) [112]. However, achievement of SVR reduces but does not eliminate the risk of HCC, particularly in patients with advanced chronic liver disease [113,114], and HCC may be observed up to ten years after eradication of HCV [113].

Recently, direct acting antivirals have changed the treatment of HCV and are associated with >90% rates of SVR [115,116,117,118]. Importantly, these drugs are virtually free of severe side effects and are being prescribed also in patients with cirrhosis, including those who are decompensated (i.e., in whom previous treatment with IFN was contraindicated) [3].

There is strong evidence by large, observational multicenter studies that treatment with DAAs is associated with a significant reduction in the risk of HCC [26,119,120]. In a study by Kanwal et al. including 22,500 patients with chronic hepatitis C, the risk of HCC was 0.9 vs. 3.4 HCC per 100 person-years in those who achieved SVR vs. those who did not [119]. Importantly, DAA-induced SVR reduces the risk of HCC in both patients with and without cirrhosis [26], though the latter group remains at risk of HCC despite virological cure [121].

As previously mentioned, IFN was associated with significant side effects and was rarely used in patients with compensated cirrhosis (and was contraindicated in patients with decompensated liver disease). By contrast, as DAAs are being used in patients with advanced fibrosis and cirrhosis, it was hypothesized that the risk of HCC in patients with DAAs-driven SVR would have been higher than that in patients with IFN-driven SVR. However, in a random-effects meta-analysis, Waziry et al. [122] find similar risks of occurrence and recurrence of HCC in patients treated by DAA vs. IFN. Importantly, these findings were confirmed by meta-regression adjustment for age and duration of follow up [122]. Comparable results were confirmed by Ioannou et al. [26] in another large cohort including 21,498 patients with chronic hepatitis C from the Veterans Administration Database.

In recent years, there has been an intense debate regarding the hypothesis that the achievement of SVR by DAAs could be associated with increased risk of HCC recurrence due to deregulation of intra-hepatic immune surveillance [123,124]. However, in a recent meta-analysis of individual patient data including 977 patients with HCV-related cirrhosis and HCC in complete response after surgical/locoregional treatments, treated with DAAs who were compared with controls from ITA.LI.CA cohort (*n* = 328, DAA-unexposed patients), there was no difference in recurrence rate between groups (RR for exposure to DAAs = 0.64, 95% CI 0.37 to 1.1; *p* = 0.1) [125].

In conclusion, eradication of HCV is associated with a significant reduction in the risk of HCC, independently of baseline severity of liver disease. However, patients with more advanced chronic liver disease remain at risk for HCC and must continue surveillance after achievement of SVR [3].

Therefore, there is a strong need for methods to assess individual patient risk. In a recent prospective multicenter study including 1054 patients with advanced fibrosis (stiffness >10 kPa) or cirrhosis (58% of the total) with a minimum of a six-month follow-up after DAAs, Ampuero et al. investigated the predictors of HCC [126]. HCC was observed in 56 patients (5.3%) after therapy and was independently predicted by Fibrosis-4 (FIB-4) > 3.25 (hazard ratio [HR] 2.26 [1.08–4.73]; *p* = 0.030), liver stiffness by transient elastography (HR 1.02 [1.00–1.04]; *p* = 0.045) and baseline cirrhosis by ultrasound (HR 3.15 [1.36–7.27]; *p* = 0.007). Interestingly, a baseline stiffness > 10 kPa or presence of cirrhosis identified patients at higher risk for development of HCC in presence of FIB-4 > 3.25 (8.8%; 44/498) versus FIB-4 < 3.25 (2.4%; 12/506), while patients with only FIB-4 > 3.25 had no HCC (0%; 0/50) (*p* = 0.0001). More interestingly, a combination of baseline FIB-4 > 3.25 and HCC screening criteria (cirrhosis or liver stiffness > 10 kPa) had an annual incidence > 1.5 cases per 100 person-years, while the patients lacking these predictors remained at <1 case. Patients who maintained post-treatment FIB-4 > 3.25 and were either cirrhotics or had baseline liver stiffness >10 kPa remained at the highest risk of HCC occurrence (13.7% [21/153] vs. 4.9% [9/184]; logRank 7.396, *p* = 0.007) [126].

It is likely that additional factors, such as alcohol consumption or non-alcoholic fatty liver disease may influence the risk of HCC in these patients [127]. Further studies are required to identify risk factors associated with higher risks for HCC development and may help improve screening strategies in patients with cirrhosis after SVR [128,129].

## 6. Immunotherapy in HCC: Does Viral Etiology Play a Role?

In the pivotal SHARP trial, as well as in subsequent HCC trials with check points inhibitors, patients with HCV-related HCC had the best survival gain [130]. Immunotherapy is now gaining continued attraction in treatment of different types of cancers [131]. Immune checkpoint molecules are central in maintaining immune tolerance and programmed cell death 1 (PD-1) and cytotoxic-T-lymphocyte-associated protein 4 (CTLA-4) have strongly emerged in immuno-oncology for their role as therapeutically actionable drivers of immune escape [132].

The combination of the immune-checkpoint inhibitors (ICIs) atezolizumab (an anti-PD-L1 antibody) and the anti-VEGFA antibody bevacizumab has produced superior results when compared with sorafenib in patients with advanced-stage HCC, setting a new first-line benchmark median overall survival (OS) duration of 19 months, thus appearing as a breakthrough in the management of this disease [133]. Similarly, the anti-PD-1 antibody sintilimab combined with a bevacizumab biosimilar (IBI305) has been reported to improve OS in Chinese patients with advanced-stage hepatitis B virus (HBV)-associated HCC relative to sorafenib [134].

One of the key principles of immunotherapy is the recognition of tumor antigens by the immune system as foreign [135]. Indeed, in the tumor microenvironment, the expression of PD-L1 in antigen-presenting cells and tumor cells is up-regulated with chronic exposure to antigens like HBV accelerating the oncogenic processes.

In the case of viral-driven HCC, the recognition of viral-related antigens or the antigens derived from the viral-induced mutations could then serve as a basis for immunotherapy.

Hepatitis viral infection disrupts normal signaling pathways; leads to sustained hepatic inflammation, fibrosis, and aberrant hepatocyte regeneration; and exerts complex biological effects on the tumor microenvironment (TME).

It is still controversial whether there is a difference in clinical response rate for immune checkpoint inhibitors (ICIs) between HBV- and HCV-associated HCC. Some reported that responses occurred regardless of HCC etiology, while some others demonstrated that clinical activity was observed predominantly in uninfected or HCV-infected cohorts [136].

In a recent metanalysis of the recent published manuscripts regarding ICIs in HCC, although immunotherapy improved survival in the overall population (hazard ratio (HR) 0.77; 95% confidence interval (CI) 0.63–0.94), survival was superior in the arm of patients with HBV-related HCC (*n* = 574; *p* = 0.0008) and HCV-related HCC (*n* = 345; *p* = 0.04), but not in patients with non-viral HCC (*n* = 737; *p* = 0.39). Patients with viral etiology (HBV or HCV infection) of liver damage and HCC showed a benefit from checkpoint inhibition (HR 0.64; 95% CI 0.48–0.94) compared with patients with HCC of a non-viral etiology [137].

The microenvironment of HBV-related HCC has a strong immunosuppressive environment, which is reversed by anti-PD1 drugs. These mechanisms may explain the impact that the presence of an HBV infection had on the efficacy of ICIs. However, unlike HBV-related HCC, the function of HCV-specific CD8 +T cells did not recover after a PD-1/PD-L blockade. Mean-while, patients with HCC and chronic HCV infections were rich in Tregs and had an upregulated expression of CTLA-4 and other immunosuppressive molecules. CTLA-4 is preferentially upregulated in PD- 1 + T cells, suggesting that antigenic stimulation induces the expression of negative co-stimulatory signals that cumulatively contribute to treatment resistance. This might explain the negative interaction between the presence of HCV infection and ICI treatment efficacy in patients with HCC [138].

## 7. Conclusions and Future Directions

Epidemiology of HCC is changing and alcohol and NASH-related HCC are increasing. However, chronic viral infection due to HBV ± HDV and HCV are still a major cause of HCC worldwide. In patients with chronic viral hepatitis, antiviral therapy significantly reduces the risk of HCC, independent of baseline severity of liver disease. However, patients with cirrhosis remain at risk of HCC and must continue surveillance. In patients at risk, particularly those with cirrhosis, screening for HCC by six-month ultrasound is recommended and improves survival. In patients with chronic HBV infection, multiple scores have been validated to predict the individual patient’s risk and identify patients in whom surveillance may be discontinued. In the future, improvement in risk stratification in patients with HCV who achieve SVR by DAA is expected and may help to identify patients at higher risk in whom surveillance must be continued despite virological cure. The role of immunotherapy in patients with viral-related HCC is being explored, but first results appear promising for the treatment of viral-related HCC.

## Figures and Tables

**Table 1:** Mechanisms of hepatic carcinogenesis in patients with chronic viral infection.

	Main Mechanisms	Pathways Involved
HBV	- HDNA integrated in hepatic-cells - X protein interaction with nuclear transcription factors and transduction pathways - Aflatoxin exposure	WNT-signaling PI3K/Akt/mTOR Ras/ERK1/2 p53 (aflatoxin exposure)
HDV	- Alterations of immune response - Epigenetic changes - Oxidative stress response	TGF-β, Smad3, STAT3, NFκβ Evasion of Interferon System
HCV	- Oxidative stress response - Adaptation to microbial stress - Antigen-presenting cells	Accumulation of fatty acids-NFκβ TERT, β-catenin, p53, Rb, KEAP Increase of IFN-β, IL-1β, IL-6, TNF-α

## Data Availability

Not applicable.
